# Flavored E-Cigarette Sales Restrictions and Young Adult Tobacco Use

**DOI:** 10.1001/jamahealthforum.2024.4594

**Published:** 2024-12-27

**Authors:** Abigail S. Friedman, Michael F. Pesko, Travis R. Whitacre

**Affiliations:** 1 Department of Health Policy and Management, Yale School of Public Health, New Haven, Connecticut; 2 Department of Economics, University of Missouri, Columbia

## Abstract

**Importance:**

More than one-quarter of US residents live in states or localities that restrict sales of flavored electronic nicotine delivery systems (ENDS), often as a means to reduce youth vaping. Yet, how these policies affect young adult vaping and smoking remains unclear.

**Objective:**

To estimate the effects of ENDS flavor restrictions on ENDS use and cigarette smoking among young adults (age 18-29 years) in the US.

**Design, Participants, and Setting:**

This quasi-experimental analysis used annual survey data from the 2016 to 2023 Behavioral Risk Factor Surveillance System (BRFSS), a series of nationally- and state-representative, repeated cross-sectional surveys of noninstitutionalized civilian adults in the US. Two-way fixed-effects specifications were estimated to assess how ENDS flavor restrictions affect young adults’ use of ENDS and cigarettes. Analyses were adjusted for potential confounders including concurrent tobacco control policies, respondent demographic characteristics, and socioeconomic factors. Data analyses were conducted in November 2023 and repeated in October 2024 to incorporate newly released 2023 survey data.

**Main Outcomes and Measures:**

Current and daily use of ENDS and of cigarettes as measured in the BRFSS.

**Results:**

Balanced panel analyses of 242 154 individuals aged 18 to 29 years consistently found that state ENDS flavor restrictions were associated with statistically significant reductions in daily vaping and increases in daily cigarette smoking. Estimates were similar across robustness checks, with the main specification showing 3.6 (95% CI, −5.0 to −2.1) percentage point (ppt) reductions in daily vaping and 2.2 ppt increases in daily cigarette smoking (95% CI, 1.0 to 3.4) relative to changes in states without restrictions. These estimates are equivalent to an 80% reduction in daily vaping and 22% increase in daily smoking compared with 2018 mean rates.

**Conclusions and Relevance:**

The findings of this quasi-experimental analysis of BRFSS survey data indicate that state restrictions on flavored ENDS sales were associated with reduced vaping among young adults but may have unintentionally increased cigarette smoking, potentially offsetting public health gains. These findings suggest that alleviating the burden of tobacco-related disease will require regulatory strategies that balance reducing vaping with preventing increases in more lethal, combustible tobacco use.

## Key Points

**Question:**

How do policies restricting sales of flavored electronic nicotine delivery systems (ENDS) affect young adult vaping practices and cigarette smoking in the US?

**Findings:**

Using a cross-sectional survey dataset covering 376 963 young adults (age 18 to 29 years), this quasi-experimental analysis found that state restrictions on flavored ENDS sales were associated with a 3.6−percentage point (ppt) reduction in daily vaping as well as a 2.2 ppt increase in daily smoking relative to trends in states without restrictions.

**Meaning:**

These findings suggest that restricting flavored ENDS sales is associated with reduced vaping but increased cigarette smoking among young adults, potentially offsetting these policies’ public health benefits.

## Teaser

This quasi-experimental survey study evaluates the association of restrictions on flavored electronic nicotine delivery systems with vaping and cigarette smoking among 18- to 29-year-old individuals in the US.

## Introduction

Among young adults in the US, use of electronic nicotine delivery systems (ENDS) rose from 5.4% in 2014 to 13.6% in 2022, while combustible cigarette use fell from 17.9% to 6.5% over that same period.^1^ The mean (median) expert believes that vaping’s effect on quality-adjusted life expectancy is 37% (25%) that of smoking.^2^ Assuming that either estimate reflects true risks, then regulating ENDS to reduce vaping without increasing smoking presents a key goal for public health policy.^3^ However, a corresponding regulatory mechanism—that is, a policy that reduces ENDS use without reducing smoking cessation or increasing smoking initiation or relapse—remains unknown.

To understand the impact of these policies, researchers have increasingly used quasi-experimental analyses: methods leveraging natural experiments that the 2021 Nobel Prize in Economic Sciences^4^ recognized for enabling causal inference from observational data. In a review^5^ of quasi-experimental analyses of ENDS minimum sales ages, advertising restrictions, and/or taxes, 16 of 18 studies concluded that these policies increased conventional cigarette use (“smoking”) compared with trends in nonadopting states.

ENDS flavor restrictions offer a newer and less thoroughly evaluated regulatory tool. A scoping review^6^ of evidence on these policies’ effects identified 30 studies in May of 2022. The authors’ GRADE (Grading of Recommendations Assessment, Development, and Evaluation) analysis concluded that there was moderate-quality evidence that ENDS flavor restrictions decrease both ENDS sales and the availability of flavored ENDS products at affected retailers, low-quality evidence that they increase cigarette sales, and very low-quality evidence for increases in cigarette use. Findings of ENDS flavor restrictions associated with decreases in flavored ENDS sales and availability at affected retailers are consistent with retailer survey data on availability in stores that are subject to these policies compared with those that are not.^7^

More recent peer-reviewed quasi-experimental studies include Dove et al’s^8^ 2-way fixed-effects (TWFE) analyses of self-reported vaping among high school students in northern California, which found no statistically significant changes in current ENDS use. An industry-funded analysis^9^ of sales data assessed flavored ENDS sales prohibitions implemented by 4 states as temporary emergency responses to the late-2019 outbreak of vaping-associated lung injuries. The authors found that flavor prohibitions were associated with reduced sales of ENDS refills but increased cigarette sales. Work neither funded by nor affiliated with the industry drew similar conclusions: matching data on all US state and local flavor restrictions to retail sales data for 44 states, Friedman et al^10^ findings indicate that restricting flavored ENDS sales reduced retail sales of ENDS, especially flavored ENDS, but increased cigarette sales.

To our knowledge, no peer-reviewed quasi-experimental study has evaluated permanent flavor restrictions’ relationship to ENDS and cigarette use across multiple states. Thus, we applied a quasi-experimental research design to nationally and state-representative data from the Behavioral Risk Factor Surveillance System (BRFSS) survey, focusing on 18- to 29-year-old respondents to compare how young adults’ ENDS and cigarette use changed in states that adopted ENDS flavor restrictions compared with those that did not, before vs after those policies went into effect. Not only are transitions into daily smoking common in this age group—52% of US daily smokers aged 26 to 29 years reported first smoking daily at 18 years or older^11^—but also benefits from cessation are particularly high: survival curves for cigarette smokers who quit before age 35 years are “nearly identical” to those of never smokers.^12^ Consequently, policies that decrease habitual smoking among 18- to 29-year-old individuals may have large benefits for population health. Thus, our objective was to estimate how ENDS flavor restrictions affect young adults’ use of cigarettes and ENDS.

## Methods

 The Yale institutional review board deemed this study not to be human subjects research (No. 2000037301). Informed consent was not necessary because the study used only anonymized secondary data from the BRFSS, which has its own consenting process.

### Study Data

The BRFSS annual cross-sectional surveys provide nationally and state-representative self-reported data on demographic characteristics, health, and health behaviors for noninstitutionalized US civilians aged 18 years and older. With ENDS questions first fielded in 2016, our analyses used data from the 2016 to 2023 waves. We limited consideration to 18- to 29-year-olds to focus on young adults,^13^ and omitted 2023-wave interviews conducted in 2024 to ensure quarterly representativeness. Focal analyses limited consideration to the balanced panel of 31 states that fielded ENDS-use questions in all waves in which they were assessed (the eData in Supplement 1 provides further details).

### Outcomes

Outcomes were binary indicators for current and daily use of ENDS (“vaping”) and cigarettes (“smoking”). Responses were based on questions asking whether the respondent “now” uses the product “every day, some days, or not at all” (the eData in Supplement 1 provides furthers details).

### Exposures

BRFSS data were matched to original data on the proportion of state residents subject to state or municipal policies restricting or prohibiting sale of flavored ENDS, flavored cigars, and menthol cigarettes (the eData in Supplement 1 provides further details). Exposures of interest were a binary indicator for state restrictions on flavored ENDS sales on the first day of each respondent’s quarter of interview and a continuous variable capturing the proportion of state residents covered by only local ENDS flavor restrictions (ie, without a state law), to estimate separate effects for statewide compared with partial policy coverage. In all but the first analysis, a separate indicator was used for Maryland’s flavor restriction, which stemmed from a regulatory enforcement prioritization document (not legislation or an executive order), and unlike other states, only applied to disposable and cartridge-based ENDS, exempting the open-system devices that many adults prefer.

### Covariates

We adjusted for concurrent policies as covariates to avoid confounding when attempting to estimate how ENDS flavor policies affect use. These covariates included other product-based sales restrictions—the proportion of state residents covered by restrictions on flavored cigar sales, restrictions on menthol cigarette sales, and bans on unflavored ENDS sales at the start of the interview quarter—and the percentage of state residents whose municipality had passed but not yet reached the effective date for flavored ENDS sales restrictions, to adjust for anticipatory responses. Other state- and time-varying tobacco policy covariates included tax rates on cigarettes, cigars,^14^ and ENDS^15^ at interview; percentage of state residents covered by workplace indoor smoking and vaping laws at the start of the interview month^16^; and an indicator for whether individuals could legally purchase tobacco products given their age and their state’s legal sales age at the start of their interview month.^14^ Covariates omitted the federal “tobacco-21 law” (included in the Further Consolidated Appropriations Act, 2020) adopted on December 20, 2019, because quarter-year fixed-effects absorbed that variation. Analyses also adjusted for annual beer tax rates^17^ and indicators for medical cannabis legalization and recreational cannabis legalization, policies that may have affected tobacco use indirectly via complementary or substitutionary behaviors.

Additional covariates included respondent sociodemographic characteristics—indicators for age, sex, race/ethnicity, whether the respondent has completed 1 year or more of college, and whether the interview was conducted by cell phone (a key sampling variable). In addition, we included state and time-varying factors related to the economy (state monthly unemployment rates^18^), COVID-19 (deaths per 100 000 residents in the respondent’s state and interview month,^19^ indicators for COVID-19−related nonessential business closures in one’s state at interview^20^), and the US outbreak of vaping-associated lung injuries, which has been shown to be associated with ENDS risks perceptions^21^; ie, total state vaping-associated lung injury deaths at the start of the respondent’s interview month and binary indicators for temporary state-level restrictions on ENDS sales in response to that outbreak that were blocked or stayed (the eData in Supplement 1 provides further details).

### Statistical Analysis

We calculated sample-weighted means for all outcomes, exposures, and tobacco policy covariates, for the full sample and the balanced panel. Then, multivariable regressions estimated TWFE analyses to assess each outcome variable’s association with ENDS flavor restrictions, with separate indicators for state laws compared with partial coverage from municipal policies. This approach adjusted for state- and quarter-year fixed-effects in addition to the aforementioned covariates to capture how an outcome variable changed in states that adopted ENDS flavor restrictions vs those that did not, before vs after the flavor policies’ effective dates, without bias from common time trends or time-invariant state characteristics. Given that logistic regressions can introduce bias in analyses with large numbers of fixed-effects, we used linear regressions^22^ with robust standard errors clustered by state, the primary level of policy variation.^23^

While simplified analyses used a single indicator for all state-level flavor policies, subsequent regressions controlled for Maryland’s policy separately, then limited the sample to the 31 states that fielded vaping questions in all BRFSS waves in which they were offered. The latter analyses were first run unweighted and then sample-weighted to clarify weighting’s influence on estimates.

Two additional robustness checks were considered. To address concerns about potential unmeasured confounders related to COVID-19, the first omitted interviews conducted during the pandemic’s first 6 months (March-August 2020). The second dropped the 10 states with the highest baseline adult smoking rates (≥21% in 2016) to ensure that adopting states’ lower baseline smoking rates did not drive our estimates.

Event studies and Goodman-Bacon decompositions^24^ assess assumptions required for causal interpretation of these estimates. Specifically, staggered policy implementation can introduce bias in TWFE analyses if policy impacts differ between states or over time because comparisons between early and late adopters will not cancel out when their effect sizes differ. Goodman-Bacon decompositions disaggregated the proportion of the TWFE estimate that stemmed from each type of comparison; that is, adopters vs never-adopters, within-adopter (ie, before vs after a state adopts), or between-adopters (ie, early vs late adopters and vice versa). This disaggregation enabled empirical assessment of whether staggered policy implementation substantively biased a TWFE analysis’s results.

If Goodman-Bacon decompositions^24^ suggested substantive bias from staggered policy implementation, we re-estimated the main specification using the De Chaisemartin and D’Haultfoeuille (DCDH) approach to generate unbiased average treatment effects,^25^ following prior ENDS policy evaluations, because DCDH is both robust to that bias and valid in contexts where policies are rescinded as well as adopted, unlike many alternatives^10,26,27^ (the eMethods in Supplement 1 provides further details). Because TWFE produces a more efficient estimator than DCDH, the former provides our preferred estimates where Goodman-Bacon decompositions do not indicate substantive bias. Statistical tests were 2-tailed and *P* < .05 was considered statistically significant. Data analyses were performed from November 2023 to October 2024 using Stata MP, version 18.0 (StataCorp).

## Results

### Sample Characteristics

**Figure 1.**
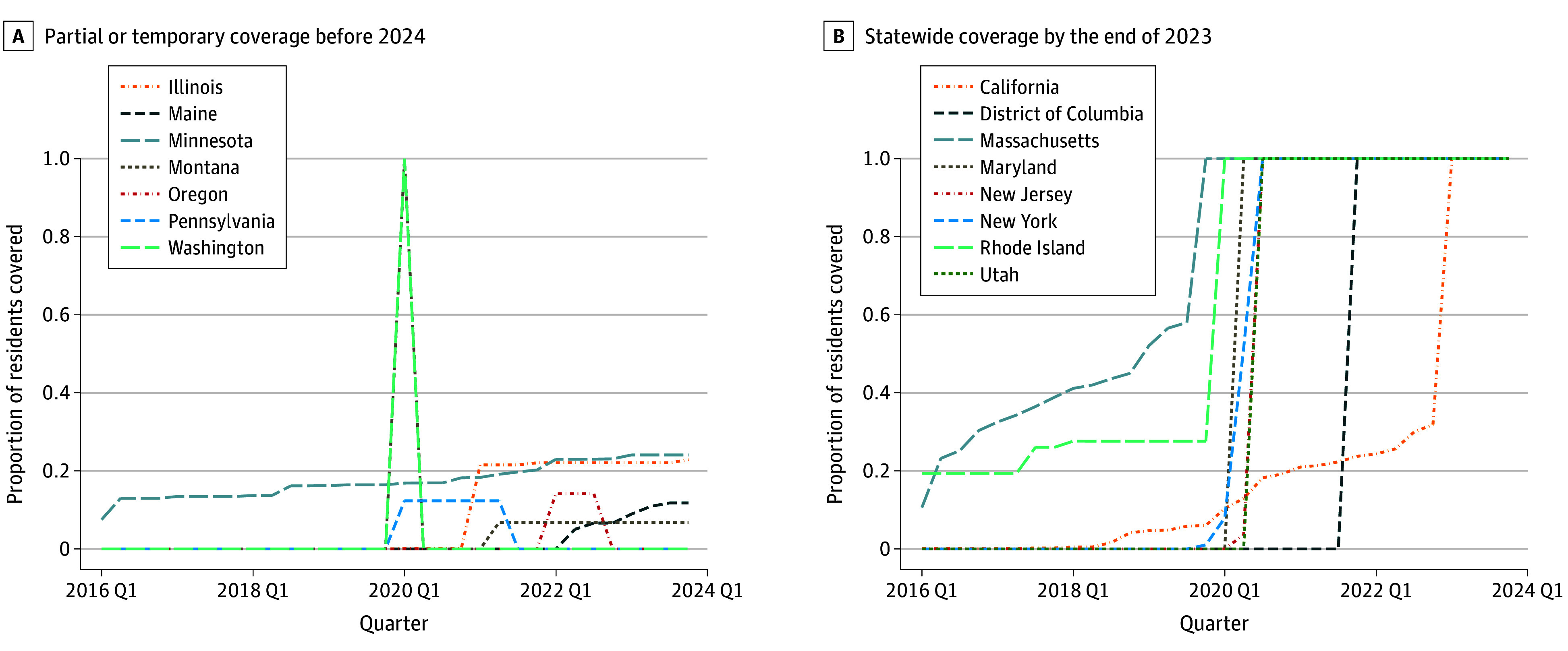
Coverage of Restrictions on Flavored Electronic Nicotine Delivery System (ENDS) Sales, by State and Quarter Proportion of state residents living in areas with a restriction on flavored ENDS sales at the start of each quarter from 2016 to 2023, for states with at least 1 quarter in which 2.5% or more of their residents were covered.

The analyses included data from 376 963 individuals aged 18 to 29 years (178 517 females [47.6%] and 198 397 males [52.6%]; 49 did not report sex [<0.1%]). The Table presents weighted means for smoking and vaping outcomes as well as policy exposures and covariates. During the analytic period, current vaping doubled and daily vaping tripled among 18- to 29-year-old individuals, whereas current and daily cigarette smoking dropped by more than half. Although no state restrictions on flavored ENDS sales were in effect in 2016, 22% of the balanced sample’s respondents were covered by a restriction in 2023. Figure 1 plots the percentage of state residents covered by restrictions on flavored ENDS sales for states covering more than 2.5% of residents on the first day of any quarter from 2016 through 2023.

**Table.** Summary Statistics of Behavioral Risk Factor Surveillance System (2016-2023 Waves) Respondents Aged 18 to 29 Years^a^

Variable	%			
	2016-2023		2016	2023
	Full data	Balanced panel	Balanced panel	Balanced panel
Participants, No.	376 963	242 154	29 177	28 665
Outcome variables				
Current ENDS use	13.4	13.6	8.4	16.9
Daily ENDS use	5.9	6.0	2.5	9.5
Current cigarette use	12.1	12.5	16.4	6.9
Daily cigarette use	7.1	7.4	10.2	3.5
Any current use (cigarettes/ENDS)	19.5	20.3	21.1	20.5
Flavor restrictions				
State restriction on flavored ENDS sales	8.8	10.6	0	22.2
Covered by substate restrictions, only on flavored ENDS sales	2.6	2.1	1.5	1.0
Flavored cigar sales restriction coverage	8.2	9.9	7.4	10.9
Menthol cigarette sales restriction coverage	5.5	5.1	0.2	10.1
Proportion of residents covered by ban on unflavored ENDS sales	0.4	0.1	0	0
Proportion of residents in interim between passage and effective date of ENDS flavor policy	1.0	0.9	0.2	0.9
Other tobacco/nicotine policies, $/mL				
ENDS tax rate, closed system products	0.43	0.26	0.09	0.50
ENDS tax rate, open system products	0.42	0.25	0.09	0.44
Cigar tax rates, $/cigar	0.01	0.20	0.19	0.20
Cigar tax rates, percentage of cost	31.8	31.4	30.5	32.0
Cigarette tax rates, $/pack	1.94	1.84	1.76	1.88
Requiring smoke-free worksites	74.7	64.2	61.6	64.4
Requiring vape-free worksites	42.7	34.0	20.7	45.1
Respondents of legal age to purchase tobacco	88.0	89.8	99.5	75.7

Abbreviation: ENDS, electronic nicotine delivery system.

^a^ The sample-weighted means were derived from data on 18- to 29 year-old individuals interviewed in the 2016 to 2023 survey waves with interview dates before 2024 (including 2018 and 2020 e-cigarette modules), matched to policy variables. Coverage variables refer to the percentage of state residents covered by a particular policy. The balanced panel was limited to the 31 states that provided data on vaping in all years: Alaska, Arkansas, Connecticut, Delaware, Georgia, Hawaii, Idaho, Indiana, Kansas, Massachusetts, Maryland, Maine, Michigan, Minnesota, Missouri, Mississippi, Montana, North Carolina, North Dakota, Nebraska, New Hampshire, New York, Ohio, Oregon, Rhode Island, South Dakota, Tennessee, Texas, Utah, Virginia, and Wyoming. In 2018, balanced panel rates of current vaping, daily vaping, current smoking, and daily smoking were 12.7%, 4.5%, 15.4%, and 10.0%, respectively.

Coverage from state and local restrictions on menthol cigarette sales and flavored cigar sales also rose during this period, as did cigarette and ENDS tax rates and clean air coverage. Thus, quasi-experimental analyses adjusting for related policies are needed to distinguish ENDS flavor restrictions’ relationship to young adult vaping and smoking from other policies’ effects.

### Analyses

#### ENDS Use

**Figure 2.**
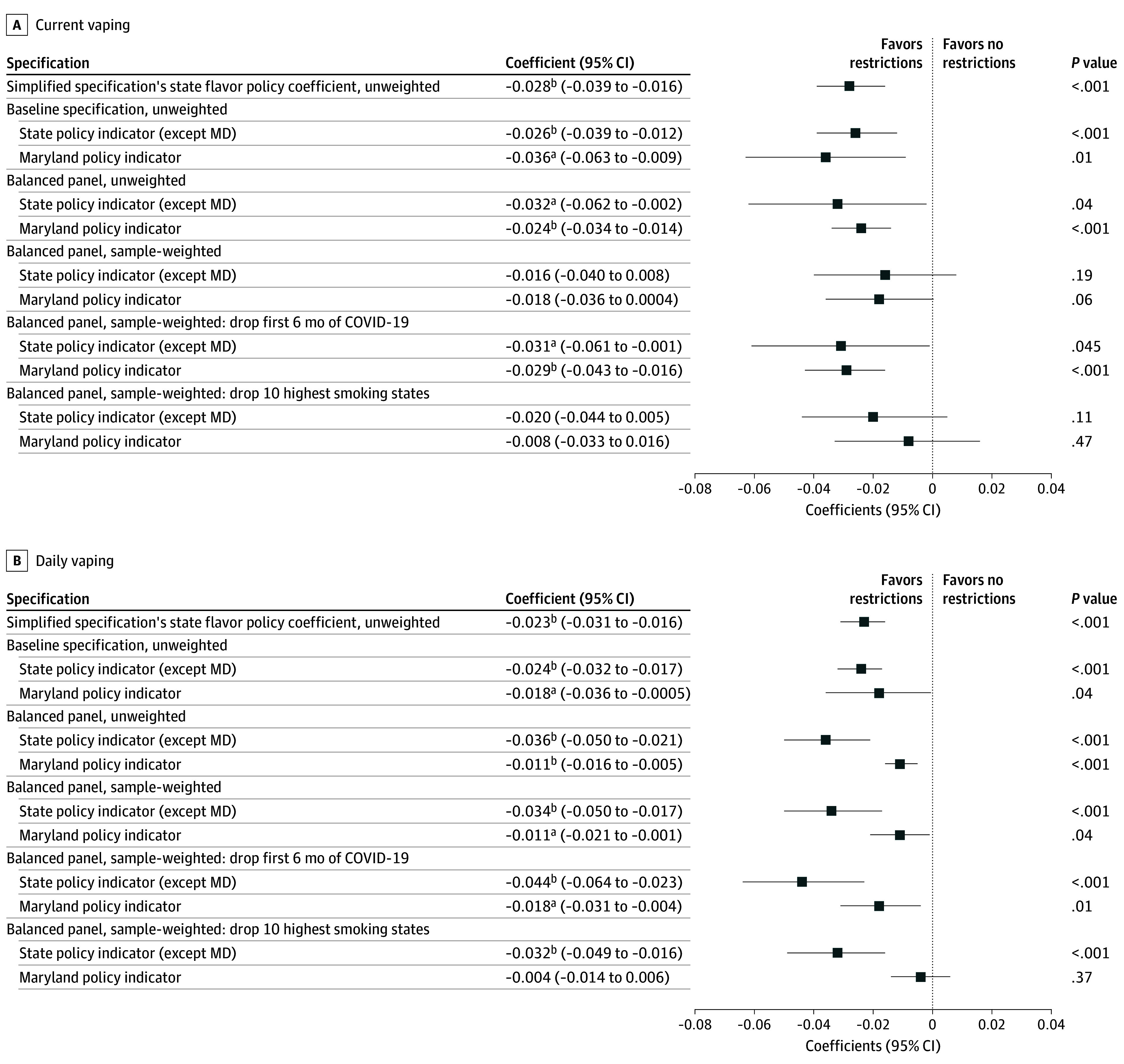
Two-Way Fixed-Effects Estimations of Electronic Nicotine Delivery System (ENDS) Flavor Restrictions and Current and Daily Vaping Among Young Adults (Age 18-29 Years) Data come from 18- to 29-year-old respondents to the 2016 to 2023 Behavioral Risk Factor Surveillance System. Forest plots depict state ENDS flavor policy coefficients and 95% CIs from a series of 2-way fixed effects regression specifications. The simplified specification has a single state ENDS flavor policy coefficient for all states, whereas all others separate out Maryland’s policy. eTable 1 in Supplement 1 provides the corresponding regression output in tabular form, along with coefficients on other tobacco and nicotine policy variables, with additional covariates described in the table notes. Robust standard errors are clustered by state. ^a^*P* < .05. ^b^*P* < .01.

Figure 2 presents TWFE estimates of ENDS flavor restrictions’ associations with current and daily vaping among young adults. Simplified specifications show statistically significant declines in current vaping (−2.8; 95% CI, −3.9 to −1.6 percentage points [ppts]) and daily vaping (−2.3; 95% CI, −3.1 to −1.6 ppts). Separating Maryland’s flavor restriction generated similar estimates for the other states’ flavor policies: −2.6 ppts for current vaping (95% CI, −3.9 to −1.2) and −2.4 ppts for daily vaping (95% CI, −3.2 to −1.7) as well as significant reductions associated with Maryland’s policy. These coefficients remain significant when limiting consideration to the balanced sample but become nonsignificant for current vaping when sample-weighted. Unweighted and weighted specifications found that state ENDS flavor restrictions were associated with daily vaping reductions of 3.6 ppts (95% CI, −5.0 to −2.1) and 3.4 ppts (95% CI, −5.0 to −1.7), respectively. In both cases, Maryland’s policy was associated with a 1.1-ppt decrease (unweighted 95% CI, −1.6 to −0.5; weighted 95% CI, −2.1 to −0.1). Implications were similar for robustness checks that dropped the first 6 months of the COVID-19 pandemic or the 10 states with the highest baseline smoking rates, although Maryland’s coefficient became nonsignificant in the latter case.

Critically, event studies were not consistent with parallel trends for current or daily vaping in the full sample specification (eFigure 1 in Supplement 1), likely due to lapses in vaping data collection. Both event studies were consistent with parallel trends when limiting consideration to the balanced panel (eFigure 2 in Supplement 1). Because Goodman-Bacon decompositions suggested that staggered adoption did not bias balanced panel coefficients (eTable 2 in Supplement 1), balanced panel TWFE estimates provided the preferred—that is, most efficient and plausibly causal—estimate for daily vaping. For weighted and unweighted regressions, these estimates represent a 76% to 80% reduction in daily vaping relative to that subsample’s rate in the year before the first statewide ENDS flavor restriction (4.5% in 2018).

#### Cigarette Use

**Figure 3.**
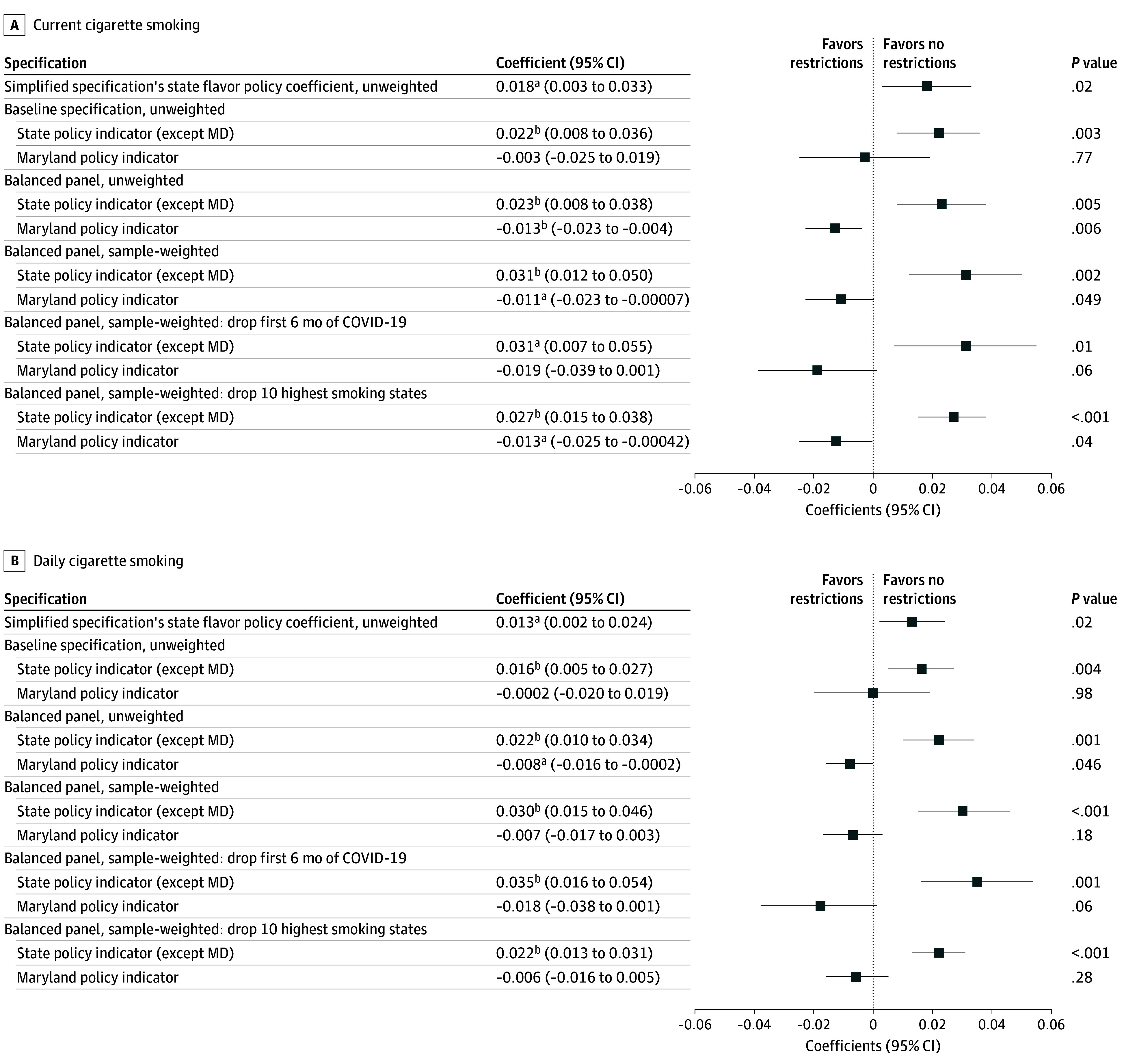
Two-Way Fixed-Effects Estimations of Electronic Nicotine Delivery System (ENDS) Flavor Restrictions and Current and Daily Cigarette Smoking Among Young Adults (Age 18-29 Years) Data come from 18- to 29-year-old respondents to the 2016 to 2023 Behavioral Risk Factor Surveillance System. Forest plots depict state ENDS flavor policy coefficients and 95% CIs from a series of 2-way fixed effects regression specifications. The simplified specification has a single state ENDS flavor policy coefficient for all states, whereas all others separate out Maryland’s policy. eTable 3 in Supplement 1 provides the corresponding regression output in tabular form, covering the aforementioned coefficients as well as coefficients on other tobacco and nicotine policy variables, with additional covariates described in the table notes. Robust standard errors are clustered by state. ^a^*P* < .05. ^b^*P* < .01

Figure 3 presents TWFE estimates of ENDS flavor restrictions’ associations with current and daily smoking among 18- to 29-year-old individuals. The simplified specifications suggest that these policies yielded increases of 1.8 (95% CI, 0.3 to 3.3) ppts and 1.3 (95% CI, 0.2 to 2.4) ppts in current and daily smoking. Similar implications and slightly larger point estimates were found when we separated Maryland’s policy and limited consideration to the balanced sample, across all robustness checks. Flavor policy point estimates for unweighted and weighted balanced sample analyses are within each other’s confidence intervals, with increases of 2.3 (95% CI, 0.8 to 3.8) ppts and 3.1 (95% CI, 1.2 to 5.0) ppts, respectively, for current smoking, and increases of 2.2 (95% CI, 1.0 to 3.4) ppts and 3.0 (95% CI, 1.5 to 4.6) ppts for daily smoking. These coefficients are equivalent to a 15% to 20% increase in current smoking and a 22% to 30% increase in daily smoking over the sample’s 2018 means (15.4% and 10.0% respectively). This specification passed both assumption tests: event studies were consistent with parallel trends for both current and daily smoking (eFigure 3 in Supplement 1), and Goodman-Bacon decompositions suggested that staggered adoption did not bias these coefficient estimates (eTable 4 in Supplement 1). Indeed, DCDH analyses yield even larger, positive point estimates, further suggesting that bias from heterogeneous effects did not drive the direction of the TWFE estimates (eFigure 4 in Supplement 1).

## Discussion

This study found that ENDS flavor restrictions were associated with decreases in vaping but marked increases in cigarette smoking among 18- to 29-year-old individuals compared with the trends expected without restrictions. Comparing effect estimates for restrictions outside Maryland suggests that, in the most conservative case, state restrictions on flavored ENDS sales yield 3.1 to 4.4 additional daily smokers for every 5 fewer daily vapers (unweighted, 2.2 ppt ÷ −3.6 ppt = 0.61; 0.61 × 5 = 3.1; weighted: 3.0 ÷ −3.4 = 0.88; 0.88 × 5 = 4.4). While these point estimates may seem small at first glance, they represent a 22% to 30% increase in daily smoking and a 76% to 80% reduction in daily vaping compared with young adults’ rates in 2018, 1 year before the first state-level restriction on flavored ENDS sales went into effect.

These findings concur with a growing body of evidence that ENDS and cigarettes are economic substitutes among youth,^27,28,29,30,31,32,33,34^ implying that policies that make ENDS more expensive (taxes) or less appealing (flavor restrictions) are likely to increase use of more dangerous combustible cigarettes in this age group. These findings reinforce the need to consider young adults as a high-priority group when developing tobacco and nicotine policies.^28,29^

Although our findings will disappoint advocates of aggressive ENDS flavor restrictions, the findings regarding Maryland’s policy suggest an alternative. Specifically, Maryland’s restriction on nonmenthol flavors in disposable and cartridge products was associated with reductions in both vaping and smoking. Because that policy exempts the open-system ENDS used more by adults than youth, it may offer a better target for interventions to reduce youth use without impeding adult smokers’ substitution away from combustible cigarettes. Or perhaps exempting menthol ENDS dampened cross-product substitution, so that those who vaped flavors and did not want to quit were nudged toward vaping menthol instead of smoking cigarettes.

Until mid-2024, the US Food and Drug Administration had not authorized marketing for any nontobacco flavored ENDS, a track record that shifted in June of 2024 with the approval of 4 menthol ENDS submitted by NJOY (Altria Group; Scottsdale, Arizona). Although flavored ENDS remain widely accessible, it is unclear whether this pattern of marketing authorizations is paving a path toward policy outcomes more similar to those estimated by this analysis for Maryland vs other states’ flavor restrictions. Future research should further investigate the potential of ENDS flavor restrictions that exempt open-system devices and/or menthol to reduce young adult vaping without increasing cigarette smoking.

### Strengths and Limitations

Our study offers several strengths over prior work. First, to our knowledge, this is the first analysis using nationally representative survey data to estimate how permanent flavor restrictions implemented across multiple states affected both smoking and vaping for young adults. Second, these analyses leveraged a uniquely detailed dataset of state and local flavored ENDS policies to adjust for municipal policies. Given that local policies were widely adopted in several states before statewide adoption, omitting these controls would have biased state policy coefficients.

Primary limitations stem from reliance on self-reported data and gaps in BRFSS data coverage for vaping. Reassuringly, while self-reported data may introduce social desirability bias, restrictions on flavored ENDS sales would need to increase reporting of cigarette use to explain our findings. It is not clear why this would be true, particularly in an anonymized survey. Unfortunately, the lack of more detailed questions regarding frequency of cigarette and ENDS use precludes distinguishing changes in experimentation from shifts in frequent but nondaily use. More detailed data are needed to clarify those relationships. Similarly, these data did not allow us to distinguish specific mechanisms driving observed relationships, such as retailer compliance, changes in manufacturer behaviors, and/or changes in ENDS risk perceptions.

## Conclusions

This study’s quasi-experimental analyses found that state restrictions on flavored ENDS sales were associated with decreased vaping but increased cigarette smoking among young adults. However, the increase in smoking was not evident for Maryland’s policy, which exempted menthol ENDS and the open-system devices used more by older adults. Understanding what drove the difference in Maryland’s response—whether a specific policy detail or contextual factors—will be important to inform regulation going forward, so that efforts to reduce vaping do not inadvertently harm public health by increasing smoking. These findings offer important considerations for US regulatory debates as state legislators consider adopting restrictions on flavored ENDS sales.

## Notes

Supplement 1. eData eMethods eFigure 1. TWFE Event Studies of ENDS Flavor Restriction Effects on Vaping eFigure 2. TWFE Event Studies of ENDS Flavor Restriction Effects on Vaping, Balanced Panel Specifications eFigure 3. TWFE Event Studies of ENDS Flavor Restriction Effects on Cigarette Smoking eFigure 4. DCDH Event Studies for State Flavor Restrictions’ Effects on Current and Daily Cigarette Smoking eTable 1. TWFE Estimates of ENDS Flavor Restriction Effects on Current and Daily Vaping eTable 2. Goodman-Bacon Decompositions for Current and Daily Vaping eTable 3. TWFE Estimates of ENDS Flavor Restriction Effects on Current and Daily Cigarette Smoking eTable 4. Goodman-Bacon Decompositions for Current and Daily Cigarette Smoking

Supplement 2. Data Sharing Statement
