# Supplementary material for: Flavored E-Cigarette Sales Restrictions and Young Adult Tobacco Use
Source: JAMA Health Forum. 2024 Dec 27;5(12):e244594. doi: 10.1001/jamahealthforum.2024.4594 (PMC11681375; doi:10.1001/jamahealthforum.2024.4594)
Supplement: Supplement 1. — eData eMethods eFigure 1. TWFE Event Studies of ENDS Flavor Restriction Effects on Vaping eFigure 2. TWFE Event Studies of ENDS Flavor Restriction Effects on Vaping, Balanced Panel Specifications eFigure 3. TWFE Event Studies of ENDS Flavor Restriction Effects on Cigarette Smoking eFigure 4. DCDH Event Studies for State Flavor Restrictions’ Effects on Current and Daily Cigarette Smoking eTable 1. TWFE Estimates of ENDS Flavor Restriction Effects on Current and Daily Vaping eTable 2. Goodman-Bacon Decompositions for Current and Daily Vaping eTable 3. TWFE Estimates of ENDS Flavor Restriction Effects on Current and Daily Cigarette Smoking eTable 4. Goodman-Bacon Decompositions for Current and Daily Cigarette Smoking [file jamahealthforum-e244594-s001.pdf]

Supplemental Online Content

Friedman AS, Pesko MF, Whitacre TR. Flavored E-Cigarette Sales Restrictions and Young Adult Tobacco Use. *JAMA Health Forum.* Published online December 27, 2024. doi:10.1001/jamahealthforum.2024.4594

eData

eMethods

eFigure 1. TWFE Event Studies of ENDS Flavor Restriction Effects on Vaping

eFigure 2. TWFE Event Studies of ENDS Flavor Restriction Effects on Vaping, Balanced Panel Specifications

eFigure 3. TWFE Event Studies of ENDS Flavor Restriction Effects on Cigarette Smoking

eFigure 4. DCDH Event Studies for State Flavor Restrictions' Effects on Current and Daily Cigarette Smoking

eTable 1. TWFE Estimates of ENDS Flavor Restriction Effects on Current and Daily Vaping

eTable 2. Goodman-Bacon Decompositions for Current and Daily Vaping

eTable 3. TWFE Estimates of ENDS Flavor Restriction Effects on Current and Daily Cigarette Smoking

eTable 4. Goodman-Bacon Decompositions for Current and Daily Cigarette Smoking

This supplemental material has been provided by the authors to give readers additional information about their work.

© 2024 Friedman AS et al. *JAMA Health Forum.*

I. eData

Analytic sample: BRFSS began fielding questions about e-cigarette use in 2016. In 2018 and 2020, these questions were included as part of an optional module, though they were dropped entirely in 2019. Thus, a "Balanced Panel" subsample limits consideration to states that fielded vaping questions in all years they were available: AK, AR, CT, DE, GA, HI, ID, IN, KS, **MA**, **MD**, ME, MI, MN, MO, MS, *MT*, NC, ND, NE, NH, **NY**, OH, OR, **RI**, SD, TN, TX, **UT**, VA, WY. States in bold adopted permanent state-level restrictions on flavored ENDS sales. Montana is italicized, as it was the only state in this sample period to implement a temporary flavor policy in effect for over 2 weeks that expired without subsequent adoption of a permanent policy. Critically, the balanced panel also omits the four states that were excluded from at least one BRFSS wave during our analytic period (NJ, FL, KY, and PA). Thus, our preferred regression specifications limit consideration to the balanced panel to ensure that all states are represented in each analytic sample wave.

Flavor restrictions: To generate a list of all states and localities with restrictions on sales of flavored tobacco or nicotine products, we reviewed multiple advocacy groups' lists and searched local and state websites.[1] For each location, we then obtained the text of municipal bills, ordinances, amendments, and/or related meeting minutes from state and local government websites or by reaching out to municipalities directly. Research assistants then reviewed each law to code policy details ranging from effective dates and products covered to retailer and product-specific exemptions. Each policy was reviewed by two research assistants, with a third "tertiary review" conducted by one or more of the authors in cases where the first two coders' entries differed, to resolve any disagreements. Policy data were then matched to state, county, and local population data from the US Census, in order to estimate the percent of state residents covered by restrictions on sales of flavored ENDS, flavored cigars, and menthol cigarettes at the start of each quarter-year, based on legislated effective dates. Corresponding measures were also calculated for flavored ENDS policy interim periods (between a restriction's passage and its legislated effective date) to capture potential anticipatory effects.

[1] Bach L. 2023. "States & Localities That Have Restricted the Sale of Flavored Tobacco Products." Campaign for Tobacco-Free Kids. July 1, 2023. https://www.tobaccofreekids.org/assets/factsheets/0398.pdf.
Truth Initiative. 2023. "Local Restrictions on Flavored Tobacco and E-Cigarette Products." Truth Initiative. April 10, 2023. https://truthinitiative.org/research-resources/emerging-tobacco-products/local-restrictions-flavored-tobacco-and-e-cigarette.

© 2024 Friedman AS et al. *JAMA Health Forum.*

Outcome variables: Indicators for current and daily ENDS use are coded based on responses to the question "Do you now use e-cigarettes, every day, some days, or not at all?" Indicators for current and daily cigarette smoking are coded based on a similar question—"Do you now smoke cigarettes, every day, some days, or not at all?"—, but this is only asked of people who first respond affirmatively to the question, "Have you smoked at least 100 cigarettes in your entire life?" Thus, individuals who have smoked <100 cigarettes in their life are coded as nonsmokers.

Covariates noted in text that may require further context:

- **Unflavored ENDS sales prohibitions:**[2] Massachusetts and 49 localities prohibited both flavored and unflavored ENDS sales at some point in 2016-2022. About a fifth of these were temporary policies, such as responses to the outbreak of vaping-associated lung injuries or regulations barring sales of ENDS that had not received FDA pre-market authorization (effectively banning all ENDS sales until the first was authorized on Oct. 12, 2021).
- **Smoke-free and vape-free workplace laws**: Data on smoke- and vape-free air laws from Seidenberg et al (2024) were expanded through the end of 2023 using data purchased from the American Nonsmokers' Rights Foundation U.S. Tobacco Control Laws Database.
- **Deaths from Vaping-Associated Lung Injuries**[3]: State-level death counts posted to CDC's website as of the first of each month, compiled for prior work.[4]
- **Blocked/Stayed ENDS Sales Restrictions adopted in response to the outbreak of vaping-associated lung injuries:** Four states had temporary bans on flavored ENDS sales in response to the outbreak of vaping-associated lung injuries that were blocked or stayed within weeks of their effective date. As those policies may reflect greater attention to the outbreak in those states, potentially impacting ENDS risk perceptions, we created indicators for interviews conducted

[2] Where all ENDS sales are prohibited, both this variable and the ENDS flavor restriction variable equal 1. Thus, the former variable's coefficient estimates the added impact from banning unflavored ENDS sales over-and-above the effect of restricting flavored ENDS sales. Summing both coefficients estimates the effect of banning all ENDS sales.
[3] While this outbreak is typically referred to as "e-cigarette or vaping product-use associated lung injury" or "EVALI" in the US, that naming has been criticized as misleading since THC products were responsible rather than nicotine e-cigarettes. (Pesko MF et al. United States public health officials need to correct e-cigarette health misinformation. *Addiction*. 2023;118(5):785-788.) To avoid confusion, we use the simplified "vaping-associated lung injuries" here, which is more consistent with Health Canada's terminology—"vaping associated lung illness" (Public Health Agency of Canada. Vaping-associated lung illness [online]. 2022. https://www.canada.ca/en/public-health/services/diseases/vaping-pulmonary-illness.html)—and used in a number of medical publications (e.g., O'Callaghan et al. Vaping-associated lung injury: a review. *Medicina*. 2022; 58(3):412.; O'Carroll et al. Vaping-associated lung injury. *Thorax* 2020;75:706-707.; etc.).
[4] Liber AC, Cahn Z, Diaz MC, Donovan E, Vallone D, Schillo B. 2023. "The EVALI Outbreak and Tobacco Sales in the USA, 2014-2020." *Tobacco Control* 32 (e2): e166–72.

© 2024 Friedman AS et al. *JAMA Health Forum.*

after each of these temporary bans was stayed but before March 1, 2020, by state. (CDC stopped collecting state case data in late-Feb. 2020.) Key dates are:

	Michigan	New York	Oregon	Utah
Effective Date	Oct. 2, 2019	Oct. 3, 2019	Oct. 15, 2019	Oct. 21, 2019
Date Ban was Blocked/Stayed	Oct. 15, 2019	Oct. 3, 2019	Oct. 17, 2019	Oct. 28, 2019

II. eMethods

Multivariable linear regressions estimate two-way fixed effects (TWFE) analyses for each outcome, with separate indicators for state policies restricting flavored ENDS sales (Flv_{isq}) vs. partial coverage from municipal policies in states without state-wide coverage (LocFlv_{isq}):

$$Y_{isq} = \beta_0 + \beta_1 \mathrm{Flv}_{isq} + \beta_2 \mathrm{LocFlv}_{isq} + \boldsymbol{\lambda}\overrightarrow{\boldsymbol{X_{isq}}} + \boldsymbol{\gamma_s} + \boldsymbol{\delta_q} + \boldsymbol{\varepsilon_{isq}} \quad \textbf{(1)}$$

Adjusting for state fixed effects ($\boldsymbol{\gamma_s}$) absorbs time invariant differences between states with more vs less flavor policy adoption. Quarter-year fixed effects ($\boldsymbol{\delta_q}$) adjust for national time-trends. The matrix $\overrightarrow{\boldsymbol{X_{isq}}}$ includes covariates described in the manuscript's methods section. Robust standard errors are clustered by state, the primary level of policy variation. [23] While simplified analyses are provided to reassure the reader that estimates are consistent across analyses, preferred specifications separate out estimation of Maryland's policy effect from the other state restrictions on flavored ENDS sales (as the former exempted open-system ENDS) and limit consideration to the "Balanced Panel" described in "Analytic Sample," above. Both unweighted and sample-weighted results are presented. Further sensitivity checks omit either the first 6 months of the COVID-19 pandemic or the 10 highest-smoking-rate states (AL, AR, IN, KY, LA, MS, MO, OH, TN, and WV).

Assumptions Required for Causal Interpretation

Causal interpretation of these analyses is predicated on three assumptions: (i) covariates adjust for other time-varying policies/events related to both the exposure and outcome; (ii) states that did versus did not adopt flavor restrictions exhibit parallel trends in their outcome variable before policies went into effect (adjusting for covariates); and, (iii) with staggered adoption, either comparisons between early-versus-late adopters do not drive effect estimates, or estimates use a method robust to dynamic treatment effects.

Including a broad range of tobacco policy covariates and adjusting for local flavor policy coverage addresses assumption (i). Event studies check for violations of parallel pre-trends to test assumption (ii), while Goodman-Bacon decompositions assess potential bias related to staggered

adoption of ENDS flavor policies (iii). If Goodman-Bacon decompositions indicate substantive bias due to staggered policy adoption, robustness checks re-estimate equation 1 using De Chaisemartin and D'Haultfœuille's (2024) approach to generate average treatment effect estimates unbiased by dynamic or heterogenous treatment effects.[5]

[5] Several methods are available to address the potential biases in staggered TWFE. To our knowledge, DCDH is the only one that allows for bidirectional changes in the exposure. This is necessary here as several ENDS flavor policies were stayed, repealed, or passed as temporary measures and allowed to expire.

© 2024 Friedman AS et al. *JAMA Health Forum.*

III. eFigures

eFigure 1: TWFE Event Studies of ENDS Flavor Restriction Effects on Vaping

A. Current Vaping

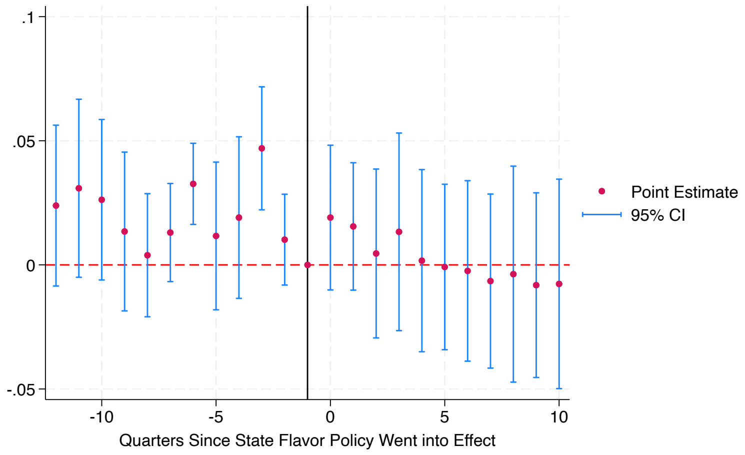

B. Daily Vaping

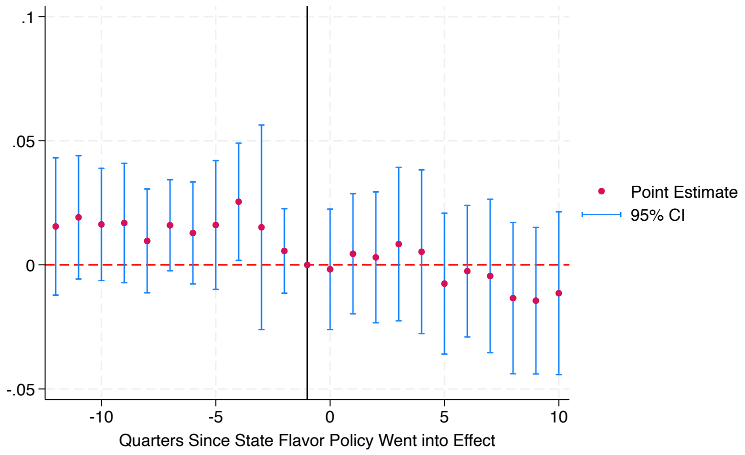

Notes: Event studies use data on 18-29 year-old participants in the 2016-2023 Behavioral Risk Factor Surveillance System surveys to assess parallel trends for the baseline specification in the full sample (eTable 1, columns 2 and 8).

© 2024 Friedman AS et al. *JAMA Health Forum.*

eFigure 2: TWFE Event Studies of ENDS Flavor Restriction Effects on Vaping, Balanced Panel Specifications

A. Current Vaping B. Daily Vaping

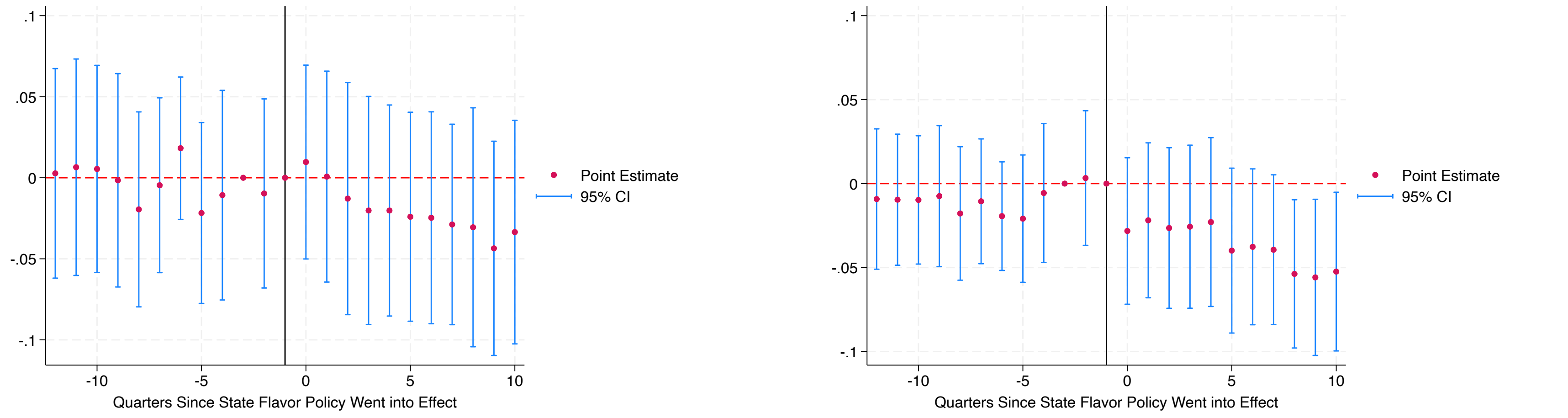

Notes: Event studies use data on 18-29 year old participants in the 2016-2023 Behavioral Risk Factor Surveillance System to assess parallel trends for the baseline specification in the balanced panel (eTable 1, columns 3 and 9).

© 2024 Friedman AS et al. *JAMA Health Forum.*

eFigure 3: TWFE Event Studies of ENDS Flavor Restriction Effects on Cigarette Smoking

A. Current Smoking B. Daily Smoking

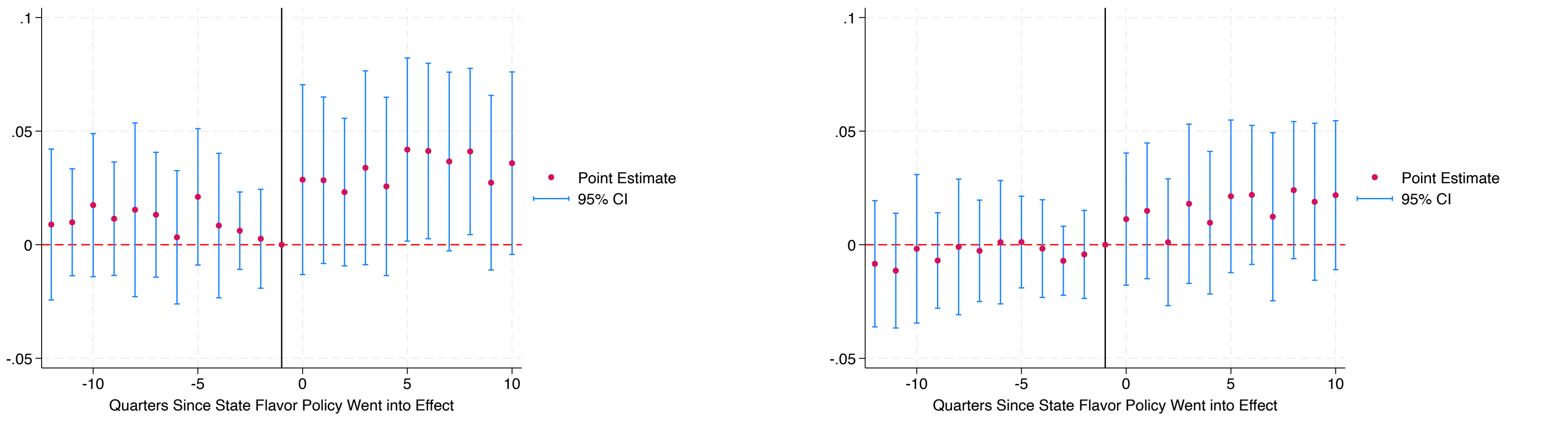

Notes: Event studies use data on 18-29 year old participants in the 2016-2023 Behavioral Risk Factor Surveillance System to assess parallel trends for the baseline specification in the balanced panel (eTable 3, columns 3 and 9).

© 2024 Friedman AS et al. *JAMA Health Forum.*

eFigure 4: DCDH Event Studies for State Flavor Restrictions' Effects on Current and Daily Cigarette Smoking

A. Current Smoking<br>ATE = 0.181 (95%CI: 0.026, 0.335)	B. Daily Smoking<br>ATE = 0.124 (95%CI: 0.016, 0.232)

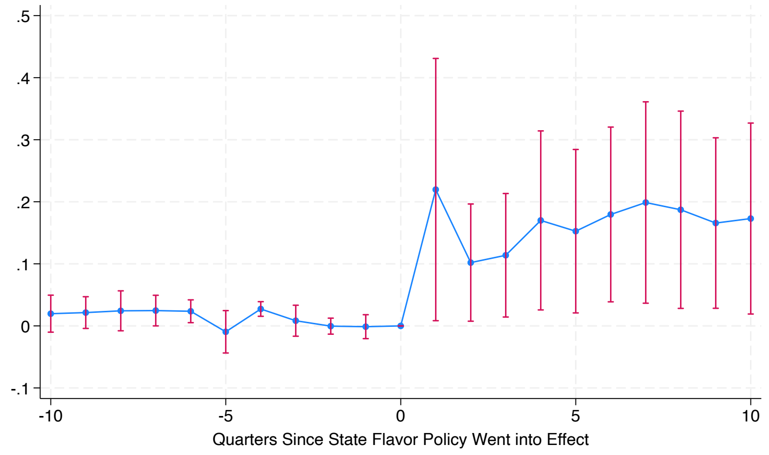

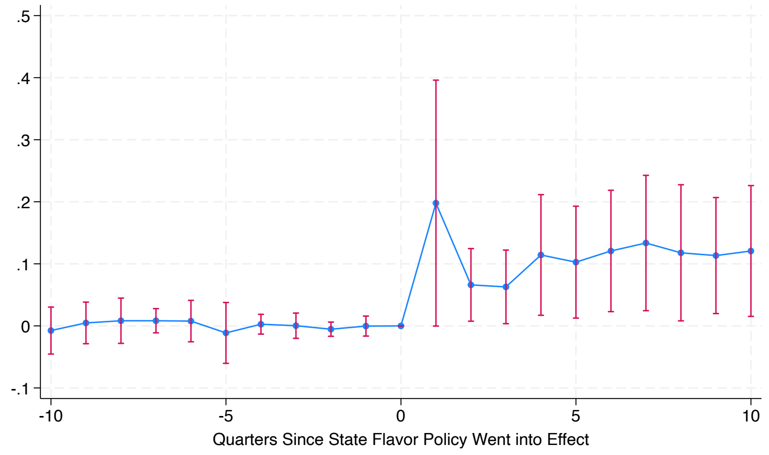

Notes: De Chaisemartin and D'Haultfœuille (2024) analyses provide two-way fixed effect estimates for the effects of state restrictions on flavored ENDS sales and corresponding event studies, robust to potential bias from heterogenous effects under staggered policy adoption. These plots are baseline balanced sample regressions adjusting for all flavor and tobacco policy covariates (diagnostic for eTable 3, columns 3 and 9). Data: 2016-2023 Behavioral Risk Factor Surveillance System for 18-29 year olds. ATE = Average Treatment Effect. 95% CI = "95% Confidence Interval"

© 2024 Friedman AS et al. *JAMA Health Forum.*

IV. eTables

eTable 1: TWFE Estimates of ENDS Flavor Restriction Effects on Current and Daily Vaping, Coefficient/[95% Confidence Interval]/p-value

	Current Vaping						Daily Vaping					
Sample‡	Full	Full	Balanced	Balanced	Balanced, drop first 6 months of Covid	Balanced, drop 10 states with highest smoking	Full	Full	Balanced	Balanced	Balanced, drop first 6 months of Covid	Balanced, drop 10 states with highest smoking
Weighted?	No	No	No	Yes	Yes	Yes	No	No	No	Yes	Yes	Yes
	(1)	(2)	(3)	(4)	(5)	(6)	(7)	(8)	(9)	(10)	(11)	(12)
State Policy Restricting Flavored ENDS sales	-0.028**						-0.023**					
	[-0.039, -0.016]						[-0.031, -0.016]					
	0.000						0.000					
Flavored ENDS Policy (except Maryland), Binary		-0.026**	-0.032*	-0.016	-0.031*	-0.020		-0.024**	-0.036**	-0.034**	-0.044**	-0.032**
		[-0.039, -0.012]	[-0.062, -0.002]	[-0.040, 0.008]	[-0.061, -0.001]	[-0.044, 0.005]		[-0.032, -0.017]	[-0.050, -0.021]	[-0.050, -0.017]	[-0.064, -0.023]	[-0.049, -0.016]
		0.000	0.039	0.194	0.045	0.114		0.000	0.000	0.000	0.000	0.000
Maryland Flavored ENDS Policy, Binary		-0.036*	-0.024**	-0.018	-0.029**	-0.008		-0.018*	-0.011**	-0.011*	-0.018*	-0.004
		[-0.063, -0.009]	[-0.034, -0.014]	[-0.036, 0.000]	[-0.043, -0.016]	[-0.033, 0.016]		[-0.036, -0.000]	[-0.016, -0.005]	[-0.021, -0.001]	[-0.031, -0.004]	[-0.014, 0.006]
		0.011	0.000	0.055	0.000	0.474		0.044	0.000	0.039	0.011	0.374
Proportion with partial Flavored ENDS Policy Coverage	-0.032	-0.031	-0.044	-0.067*	-0.096**	-0.072**	-0.029	-0.030	-0.038*	-0.053*	-0.072**	-0.045*
	[-0.070, 0.007]	[-0.069, 0.008]	[-0.106, 0.017]	[-0.121, -0.014]	[-0.150, -0.043]	[-0.118, -0.025]	[-0.060, 0.002]	[-0.061, 0.001]	[-0.071, -0.006]	[-0.094, -0.011]	[-0.116, -0.029]	[-0.089, -0.002]
	0.106	0.116	0.152	0.015	0.001	0.004	0.063	0.059	0.022	0.015	0.002	0.041
% Banning unflavored ENDS Sales	0.154	0.152	-0.659	-0.065	-0.895	0.098	-0.165	-0.164	-1.318**	-1.467**	-1.678**	-1.153*
	[-0.142, 0.450]	[-0.142, 0.445]	[-1.613, 0.295]	[-2.187, 2.056]	[-2.207, 0.417]	[-2.063, 2.259]	[-0.369, 0.038]	[-0.366, 0.039]	[-1.921, -0.714]	[-2.489, -0.444]	[-2.511, -0.844]	[-2.103, -0.203]
	0.301	0.305	0.169	0.950	0.174	0.926	0.109	0.111	0.000	0.006	0.000	0.019
% Any Cigar Flavor Policy	-0.063*	-0.062*	-0.083	-0.025	-0.065	-0.035	-0.039**	-0.040**	-0.055*	-0.039*	-0.070*	-0.041*
	[-0.113, -0.012]	[-0.112, -0.011]	[-0.194, 0.027]	[-0.080, 0.030]	[-0.147, 0.018]	[-0.094, 0.023]	[-0.065, -0.014]	[-0.066, -0.014]	[-0.109, -0.002]	[-0.075, -0.003]	[-0.125, -0.016]	[-0.071, -0.010]
	0.017	0.018	0.133	0.361	0.119	0.228	0.003	0.004	0.043	0.033	0.013	0.012
% Menthol Cigarette Policy	0.062**	0.060*	0.059	0.020	0.036	0.005	0.037**	0.039**	0.043*	0.046**	0.056**	0.041**
	[0.016, 0.108]	[0.013, 0.107]	[-0.002, 0.121]	[-0.015, 0.055]	[-0.016, 0.088]	[-0.032, 0.042]	[0.014, 0.061]	[0.014, 0.063]	[0.010, 0.076]	[0.022, 0.069]	[0.018, 0.093]	[0.020, 0.061]
	0.010	0.013	0.058	0.249	0.171	0.773	0.003	0.003	0.012	0.001	0.005	0.000
Proportion of residents in interim between ENDS flavor policy passage & effective dates	-0.021**	-0.021**	-0.016	-0.041	-0.128	-0.038	-0.019**	-0.020**	-0.023**	-0.038*	-0.117	-0.029
	[-0.035, -0.008]	[-0.034, -0.007]	[-0.037, 0.005]	[-0.121, 0.038]	[-0.267, 0.011]	[-0.138, 0.061]	[-0.032, -0.007]	[-0.032, -0.007]	[-0.035, -0.011]	[-0.072, -0.004]	[-0.238, 0.005]	[-0.064, 0.005]
	0.003	0.003	0.124	0.300	0.071	0.436	0.003	0.003	0.001	0.029	0.059	0.094
ENDS Tax Rate, Closed Systems	0.013	0.015	0.022	-0.013	0.002	-0.015	0.000	-0.001	0.008	-0.005	0.003	-0.006
	[-0.015, 0.041]	[-0.015, 0.045]	[-0.029, 0.072]	[-0.072, 0.047]	[-0.059, 0.063]	[-0.074, 0.044]	[-0.018, 0.019]	[-0.022, 0.020]	[-0.018, 0.035]	[-0.044, 0.034]	[-0.039, 0.046]	[-0.047, 0.034]
	0.351	0.318	0.387	0.673	0.946	0.608	0.989	0.917	0.525	0.809	0.872	0.750
	-0.020	-0.023	-0.025	-0.003	-0.007	0.006	-0.004	-0.002	-0.005	0.001	-0.000	0.004

© 2024 Friedman AS et al. *JAMA Health Forum.*

ENDS Tax Rate, Open Systems	[-0.044, 0.004]	[-0.050, 0.005]	[-0.064, 0.015]	[-0.054, 0.048]	[-0.056, 0.041]	[-0.046, 0.058]	[-0.019, 0.012]	[-0.021, 0.017]	[-0.027, 0.016]	[-0.031, 0.034]	[-0.034, 0.034]	[-0.031, 0.040]
	0.103	0.108	0.210	0.903	0.756	0.817	0.648	0.827	0.608	0.935	0.997	0.801
Cigar Tax Rate (% of cost)	-0.001	-0.001	-0.001	-0.000	-0.000	-0.000	-0.000	-0.000	-0.000	-0.000	-0.000	-0.000
	[-0.001, 0.000]	[-0.001, 0.000]	[-0.002, 0.001]	[-0.002, 0.002]	[-0.002, 0.001]	[-0.002, 0.002]	[-0.001, 0.000]	[-0.001, 0.000]	[-0.001, 0.000]	[-0.002, 0.001]	[-0.002, 0.001]	[-0.002, 0.001]
	0.182	0.186	0.220	0.840	0.621	0.915	0.842	0.784	0.346	0.614	0.455	0.672
Cigarette Tax ($/pack)	0.004	0.004	-0.003	0.002	-0.003	-0.001	0.001	0.001	-0.007	-0.003	-0.006	-0.003
	[-0.004, 0.012]	[-0.004, 0.012]	[-0.021, 0.016]	[-0.012, 0.016]	[-0.021, 0.015]	[-0.012, 0.011]	[-0.007, 0.008]	[-0.007, 0.008]	[-0.016, 0.002]	[-0.011, 0.004]	[-0.015, 0.004]	[-0.010, 0.004]
	0.344	0.348	0.768	0.795	0.706	0.890	0.872	0.878	0.141	0.412	0.214	0.425
Smoke-free Worksite, % Covered	-0.003	-0.003	-0.037**	-0.041**	-0.045**	-0.058**	-0.002	-0.002	-0.027**	-0.040**	-0.045**	-0.042**
	[-0.029, 0.022]	[-0.029, 0.022]	[-0.057, -0.018]	[-0.062, -0.019]	[-0.067, -0.023]	[-0.091, -0.025]	[-0.017, 0.012]	[-0.017, 0.012]	[-0.036, -0.018]	[-0.061, -0.020]	[-0.068, -0.022]	[-0.070, -0.014]
	0.788	0.790	0.000	0.001	0.000	0.001	0.768	0.765	0.000	0.000	0.000	0.005
Vape-free Worksite, % Covered	0.005	0.005	0.011	-0.005	-0.000	0.011	0.002	0.002	0.009**	0.006	0.008*	0.008
	[-0.009, 0.020]	[-0.009, 0.020]	[-0.008, 0.029]	[-0.020, 0.010]	[-0.016, 0.015]	[-0.012, 0.035]	[-0.006, 0.009]	[-0.005, 0.009]	[0.003, 0.014]	[-0.001, 0.012]	[0.000, 0.016]	[-0.006, 0.023]
	0.451	0.464	0.247	0.520	0.949	0.336	0.663	0.648	0.005	0.090	0.044	0.246
Person is of legal age to buy tobacco in their state	0.035**	0.035**	0.038**	0.038**	0.040**	0.041**	0.010**	0.010**	0.012**	0.012*	0.012*	0.012*
	[0.025, 0.045]	[0.025, 0.045]	[0.025, 0.051]	[0.021, 0.054]	[0.025, 0.056]	[0.024, 0.058]	[0.004, 0.016]	[0.004, 0.016]	[0.004, 0.021]	[0.002, 0.023]	[0.002, 0.021]	[0.000, 0.023]
	0.000	0.000	0.000	0.000	0.000	0.000	0.003	0.003	0.006	0.020	0.021	0.044
Quarter-Year & State Fixed Effects?	Yes	Yes	Yes	Yes	Yes	Yes	Yes	Yes	Yes	Yes	Yes	Yes
N	280772	280772	188454	188454	175981	157585	280772	280772	188454	188454	175981	157585
Adjusted R^2	0.036	0.036	0.035	0.035	0.034	0.034	0.030	0.030	0.029	0.032	0.032	0.031

Notes: Two-way fixed effect analyses use data on 18-29 year-old respondents to the 2016-2022 Behavioral Risk Factor Surveillance System. Covariates not noted above include respondent sociodemographic characteristics—fixed effects for year of age, gender, race/ethnicity, whether they completed a year or more of college, and whether the survey was conducted by cell phone, a key sampling variable in this survey—and non-tobacco-policy covariates: indicators for medical and recreational cannabis legalization in the respondent's state, beer tax rates, unemployment rates, COVID-19 non-essential business closures at interview, state monthly COVID-19 deaths/capita, and total deaths from the 2019 outbreak of vaping-associated lung injuries at the start of each interview month. Additionally, for states that attempted to implement emergency restrictions on ENDS sales in response to the 2019 vaping-associated lung injury outbreak but had their policy stayed by courts, a binary variable adjusts for interviews conducted between that legal stay and February 2020, the month CDC stopped collecting data on vaping-associated lung injury cases, to adjust for those policies' effects on risk perceptions. Robust standard errors are clustered by state. * p<0.05, ** p<0.01

‡The full sample includes all 18-29 year-old respondents to the 2016-2023 waves who were interviewed prior to 2024. The balanced panel sample limits consideration to those in 31 states that included vaping questions in their BRFSS survey in every wave those questions were available. This restriction omits six states that implemented a restriction on flavored ENDS sales at either the state level or municipal level with local policies covering > 1% of state residents: CA, CO, DC, IL, NJ, PA, WA. Dropping the 10 highest smoking rate states omits AL, AR, IN, KY, LA, MS, MO, OH, TN, and WV.

© 2024 Friedman AS et al. *JAMA Health Forum.*

eTable 2: Goodman-Bacon Decompositions for Current & Daily Vaping

	Current Vaping		Daily Vaping	
	Beta	Weight	Beta	Weight
A) Early vs. Later Adopters & vice-versa	-0.0122	0.0205	0.0115	0.0205
B) Adopters vs Never-adopters	-0.0527	0.4998	-0.0416	0.4998
C) Within-adopter (i.e., pre- vs. post-adoption)	-0.0143	0.4797	-0.0343	0.4797

Notes: Analyses limit consideration to the balanced sample and drop MT, as it is the only state in that panel where a temporary flavor policy was allowed to expire. (Goodman-Bacon decompositions require monotonicity in the exposure; that is, the flavor policy may either increase or decrease within a state, not both.) These decompositions disaggregate the components driving a coefficient estimate into three parts, only one of which may be subject to bias from staggered implementation in cases where effect magnitudes differ between states or over time. That component is "A" above. As implied coefficient estimates are based on weighted sums across the comparators, the numbers above enable comparison of point estimates when comparison A is included vs. omitted. Respectively, these are an increase of -3.3 vs. -3.3 ppts for current vaping, and -3.7 vs -3.7 ppts for daily vaping. Thus, potential bias from staggered adoption does not substantively alter estimates of flavor policies' relationship to current and daily vaping.

© 2024 Friedman AS et al. *JAMA Health Forum.*

eTable 3: TWFE Estimates of ENDS Flavor Restriction Effects on Current and Daily Cigarette Smoking, Coefficient/[95% Confidence Interval]/p-value

	Current Smoking						Daily Smoking					
Sample‡	Full	Full	Balanced	Balanced	Balanced, drop first 6 months of Covid	Balanced, drop 10 states with highest smoking	Full	Full	Balanced	Balanced	Balanced, drop first 6 months of Covid	Balanced, drop 10 states with highest smoking
Weighted?	No	No	No	Yes	Yes	Yes	No	No	No	Yes	Yes	Yes
	(1)	(2)	(3)	(4)	(5)	(6)	(7)	(8)	(9)	(10)	(11)	(12)
State Policy Restricting Flavored ENDS sales	0.018*						0.013*					
	[0.003, 0.033]						[0.002, 0.024]					
	0.020						0.018					
Flavored ENDS Policy (except Maryland), Binary		0.022**	0.023**	0.031**	0.031*	0.027**		0.016**	0.022**	0.030**	0.035**	0.022**
		[0.008, 0.036]	[0.008, 0.038]	[0.012, 0.050]	[0.007, 0.055]	[0.015, 0.038]		[0.005, 0.027]	[0.010, 0.034]	[0.015, 0.046]	[0.016, 0.054]	[0.013, 0.031]
		0.003	0.005	0.002	0.012	0.000		0.004	0.001	0.000	0.001	0.000
Maryland Flavored ENDS Policy, Binary		-0.003	-0.013**	-0.011*	-0.019	-0.013*		-0.000	-0.008*	-0.007	-0.018	-0.006
		[-0.025, 0.019]	[-0.023, -0.004]	[-0.023, -0.000]	[-0.039, 0.001]	[-0.025, -0.000]		[-0.020, 0.019]	[-0.016, -0.000]	[-0.017, 0.003]	[-0.038, 0.001]	[-0.016, 0.005]
		0.773	0.006	0.049	0.058	0.043		0.983	0.046	0.181	0.060	0.278
Proportion with partial Flavored ENDS Policy Coverage	0.026	0.029	0.020	0.038*	0.026	0.032*	0.017	0.019	0.009	0.033*	0.015	0.022
	[-0.008, 0.060]	[-0.005, 0.062]	[-0.015, 0.056]	[0.001, 0.074]	[-0.007, 0.059]	[0.005, 0.058]	[-0.019, 0.052]	[-0.017, 0.055]	[-0.012, 0.030]	[0.005, 0.062]	[-0.015, 0.044]	[-0.007, 0.051]
	0.126	0.091	0.253	0.042	0.113	0.020	0.342	0.301	0.411	0.023	0.318	0.136
% Banning unflavored ENDS Sales	0.015	0.011	0.004	-0.000	-0.004	-0.010	0.004	0.002	-0.012	-0.013	-0.025	-0.019*
	[-0.010, 0.039]	[-0.011, 0.034]	[-0.016, 0.023]	[-0.026, 0.026]	[-0.032, 0.024]	[-0.028, 0.009]	[-0.027, 0.035]	[-0.028, 0.032]	[-0.027, 0.003]	[-0.034, 0.008]	[-0.052, 0.001]	[-0.034, -0.004]
	0.228	0.327	0.692	0.994	0.758	0.303	0.787	0.902	0.114	0.213	0.062	0.016
% Any Cigar Flavor Restriction	0.050**	0.050*	0.039	0.049*	0.059*	0.041*	0.053**	0.053**	0.051**	0.067**	0.079**	0.050**
	[0.014, 0.085]	[0.012, 0.089]	[-0.016, 0.093]	[0.002, 0.096]	[0.004, 0.114]	[0.004, 0.079]	[0.026, 0.080]	[0.025, 0.082]	[0.014, 0.089]	[0.034, 0.100]	[0.040, 0.119]	[0.027, 0.073]
	0.007	0.011	0.155	0.044	0.035	0.032	0.000	0.000	0.009	0.000	0.000	0.000
% Menthol Cigarette Restriction	-0.026	-0.029	-0.027	-0.014	-0.041	-0.011	-0.033*	-0.035*	-0.032*	-0.035	-0.059*	-0.035**
	[-0.066, 0.013]	[-0.070, 0.012]	[-0.060, 0.006]	[-0.063, 0.035]	[-0.098, 0.015]	[-0.039, 0.017]	[-0.063, -0.004]	[-0.065, -0.005]	[-0.059, -0.004]	[-0.074, 0.005]	[-0.105, -0.013]	[-0.055, -0.015]
	0.189	0.162	0.105	0.555	0.145	0.440	0.029	0.025	0.025	0.086	0.013	0.001
Proportion of residents in interim between ENDS flavor policy passage & effective dates	0.005	0.006	0.006	0.017	0.048*	0.010	0.003	0.003	0.007	0.029	0.036*	0.023
	[-0.010, 0.020]	[-0.008, 0.020]	[-0.015, 0.026]	[-0.003, 0.038]	[0.011, 0.085]	[-0.009, 0.030]	[-0.010, 0.016]	[-0.009, 0.016]	[-0.011, 0.026]	[-0.002, 0.060]	[0.002, 0.070]	[-0.008, 0.055]
	0.488	0.404	0.569	0.096	0.012	0.285	0.650	0.591	0.418	0.066	0.038	0.144
	0.002	0.007	0.045**	0.041	0.058*	0.038	0.005	0.009	0.032**	0.044*	0.058*	0.041*

© 2024 Friedman AS et al. *JAMA Health Forum.*

ENDS Tax Rate, Closed Systems	[-0.019, 0.023]	[-0.020, 0.034]	[0.020, 0.070]	[-0.007, 0.088]	[0.006, 0.110]	[-0.003, 0.080]	[-0.013, 0.024]	[-0.015, 0.032]	[0.011, 0.053]	[0.003, 0.084]	[0.013, 0.104]	[0.006, 0.077]
	0.862	0.594	0.001	0.091	0.031	0.070	0.583	0.459	0.005	0.035	0.014	0.025
ENDS Tax Rate, Open Systems	0.003	-0.003	-0.039**	-0.043*	-0.056*	-0.042*	-0.002	-0.006	-0.030**	-0.049*	-0.063**	-0.044*
	[-0.016, 0.021]	[-0.028, 0.021]	[-0.058, -0.020]	[-0.084, -0.002]	[-0.101, -0.010]	[-0.079, -0.004]	[-0.018, 0.014]	[-0.027, 0.015]	[-0.047, -0.013]	[-0.087, -0.011]	[-0.106, -0.020]	[-0.078, -0.011]
	0.755	0.780	0.000	0.040	0.018	0.033	0.809	0.571	0.001	0.013	0.005	0.012
Cigar Tax Rate (% of cost)	-0.000	-0.000	-0.001**	-0.001	-0.001	-0.001	-0.000	-0.000	-0.001**	-0.001	-0.001*	-0.001*
	[-0.001, 0.000]	[-0.001, 0.001]	[-0.002, -0.000]	[-0.002, 0.000]	[-0.003, 0.000]	[-0.002, 0.000]	[-0.001, 0.000]	[-0.001, 0.000]	[-0.001, -0.000]	[-0.002, 0.000]	[-0.003, -0.000]	[-0.002, -0.000]
	0.474	0.651	0.002	0.124	0.057	0.122	0.291	0.384	0.008	0.052	0.034	0.047
Cigarette Tax ($/pack)	-0.001	-0.000	-0.006	-0.004	-0.007	-0.008	0.001	0.001	-0.001	-0.001	-0.001	-0.005
	[-0.009, 0.007]	[-0.008, 0.007]	[-0.015, 0.003]	[-0.018, 0.010]	[-0.023, 0.008]	[-0.019, 0.003]	[-0.006, 0.007]	[-0.006, 0.007]	[-0.009, 0.006]	[-0.012, 0.010]	[-0.013, 0.011]	[-0.013, 0.003]
	0.874	0.929	0.161	0.557	0.330	0.135	0.878	0.828	0.696	0.867	0.849	0.194
Smoke-free Worksite, % Covered	0.012	0.012	0.010	0.036*	0.031	0.035	0.011	0.011	0.013*	0.026	0.023	0.010
	[-0.007, 0.031]	[-0.007, 0.030]	[-0.004, 0.024]	[0.001, 0.072]	[-0.006, 0.069]	[-0.002, 0.071]	[-0.004, 0.026]	[-0.004, 0.026]	[0.000, 0.027]	[-0.002, 0.055]	[-0.007, 0.053]	[-0.018, 0.038]
	0.218	0.217	0.149	0.046	0.093	0.060	0.135	0.134	0.048	0.071	0.121	0.460
Vape-free Worksite, % Covered	-0.005	-0.005	-0.005	-0.017*	-0.016*	-0.019	-0.002	-0.002	-0.003	-0.012	-0.011	0.002
	[-0.014, 0.004]	[-0.014, 0.004]	[-0.015, 0.004]	[-0.031, -0.003]	[-0.030, -0.002]	[-0.040, 0.003]	[-0.010, 0.006]	[-0.011, 0.006]	[-0.013, 0.007]	[-0.026, 0.003]	[-0.025, 0.004]	[-0.013, 0.017]
	0.257	0.234	0.283	0.021	0.025	0.085	0.611	0.584	0.533	0.112	0.145	0.814
Person is of legal age to buy tobacco in their state	-0.016**	-0.017**	-0.016**	-0.020**	-0.021**	-0.020**	-0.015**	-0.015**	-0.015**	-0.018**	-0.020**	-0.016**
	[-0.021, -0.011]	[-0.021, -0.012]	[-0.021, -0.010]	[-0.026, -0.014]	[-0.029, -0.012]	[-0.027, -0.012]	[-0.020, -0.011]	[-0.020, -0.011]	[-0.020, -0.011]	[-0.024, -0.012]	[-0.027, -0.013]	[-0.023, -0.009]
	0.000	0.000	0.000	0.000	0.000	0.000	0.000	0.000	0.000	0.000	0.000	0.000
Quarter-Year & State Fixed Effects?	Yes	Yes	Yes	Yes	Yes	Yes	Yes	Yes	Yes	Yes	Yes	Yes
N	356924	356924	229572	229572	213136	193303	356924	356924	229572	229572	213136	193303
Adjusted R^2	0.072	0.072	0.075	0.074	0.073	0.068	0.063	0.063	0.065	0.065	0.064	0.058

Notes: Two-way fixed effect analyses use data on 18-29 year-old respondents to the 2016-2022 Behavioral Risk Factor Surveillance System. Covariates not noted above include respondent sociodemographic characteristics—fixed effects for year of age, gender, race/ethnicity, whether they completed a year or more of college, and whether the survey was conducted by cell phone, a key sampling variable in this survey—and non-tobacco-policy covariates: indicators for medical and recreational cannabis legalization in the respondent's state, beer tax rates, unemployment rates, COVID-19 non-essential business closures at interview, state monthly COVID-19 deaths/capita, and total deaths from the 2019 outbreak of vaping-associated lung injuries at the start of each interview month. Additionally, for states that attempted to implement emergency restrictions on ENDS sales in response to the 2019 vaping-associated lung injury outbreak but had their policy stayed by courts, a binary variable adjusts for interviews conducted between that legal stay and February 2020, the month CDC stopped collecting data on vaping-associated lung injury cases, to adjust for those policies' effects on risk perceptions. Robust standard errors are clustered by state. * p<0.05, ** p<0.01

‡The full sample includes all 18-29 year-old respondents to the 2016-2023 waves who were interviewed prior to 2024. The balanced panel sample limits consideration to those in 31 states that included vaping questions in their BRFSS survey in every wave those questions were available. This restriction omits six states that implemented a restriction on flavored ENDS sales at either the state level or municipal level with local policies covering > 1% of state residents: CA, CO, DC, IL, NJ, PA, WA. Dropping the 10 highest smoking rate states omits AL, AR, IN, KY, LA, MS, MO, OH, TN, and WV.

© 2024 Friedman AS et al. *JAMA Health Forum.*

eTable 4: Goodman-Bacon Decompositions for Current & Daily Cigarette Smoking

	Current Smoking		Daily Smoking	
	Beta	Weight	Beta	Weight
A) Early vs. Later Adopters & vice-versa	0.0103	0.0492	0.0149	0.0492
B) Adopters vs Never-adopters	0.0488	0.6941	0.0472	0.6941
C) Within-adopter (i.e., pre- vs. post-adoption)	0.0466	0.2567	0.0275	0.2567

Notes: Analyses limit consideration to the balanced sample and drop MT, as it is the only state in that panel where a temporary flavor policy was allowed to expire. (Goodman-Bacon decompositions require monotonicity in the exposure; that is, the flavor policy may either increase or decrease within a state, not both.) These decompositions disaggregate the components driving a coefficient estimate into three parts, only one of which may be subject to bias due to staggered implementation in cases where effect magnitudes differ between states or over time. That component is "A" above. As implied coefficient estimates are based on weighted sums across the comparators, the numbers above enable comparison of the point estimate comparison A is included vs. omitted. Respectively, these are an increase of 4.6 vs. 4.6 ppts for current smoking, and 4.1 vs 4.0 ppts for daily smoking. Thus, potential bias from staggered adoption does not substantively alter estimates of flavor policies' relationship to current and daily cigarette smoking.

© 2024 Friedman AS et al. *JAMA Health Forum.*
