# Supplementary material for: Flavored E-Cigarette Sales Restrictions and Young Adult Tobacco Use
Source: JAMA Health Forum. 2024 Dec 27;5(12):e244594. doi: 10.1001/jamahealthforum.2024.4594 (PMC11681375; doi:10.1001/jamahealthforum.2024.4594)
Supplement: Supplement 2. — Data Sharing Statement [file jamahealthforum-e244594-s002.pdf]

Data Sharing Statement

Friedman. Flavored E-Cigarette Sales Restrictions and Young Adult Tobacco Use. *JAMA Health Forum*. Published December 27, 2024. doi:10.1001/jamahealthforum.2024.4594

Data
Data available: No

Additional Information
Explanation for why data not available: Behavioral Risk Factor Surveillance System survey data are publicly available for download (https://www.cdc.gov/brfss/data_documentation/index.htm). We do not have approval to disseminate those data separately from CDC. Our state-by-quarter flavor policy data will be made available as part of a separate manuscript currently under peer review, once that article is published.
